# Division of neuromuscular compartments and localization of the center of the intramuscular nerve-dense region in pelvic wall muscles based on Sihler's staining

**DOI:** 10.1007/s12565-023-00744-4

**Published:** 2023-09-28

**Authors:** Xiangnan Hu, Meng Wang, Xiaojiao He, Peng Chen, Fangfang Jia, Danli Wang, Shengbo Yang

**Affiliations:** 1 Department of Anatomy, Xinpu New Developing Area, Zunyi Medical University, 6 West University Road, Zunyi, 563099 People’s Republic of China; 2 Department of Radiology, The Affiliated Hospital of Zunyi Medical University, Zunyi, 563003 People’s Republic of China

**Keywords:** Intramuscular nerve, Muscle spasm, Nerve block, Neuromuscular compartment, Pelvic wall muscle

## Abstract

The innervation of the pelvic wall muscles is not very clear. This study aimed to reveal the division of neuromuscular compartments and localize the surface position and depth of the center of the intramuscular nerve-dense region (CINDR) of the pelvic wall muscles based on Sihler's staining. Twenty-four adult cadavers were used. To localize the CINDR of the pelvic wall muscles, horizontal (H) and longitudinal (L) reference lines were drawn, and Sihler’s staining was used to reveal the intramuscular nerve distribution. The CINDR projection points (P and P′ points) behind and in front of the body surface, the positions of the P points projected onto the H and L lines (P_H_ and P_L_ points), and the depth of CINDR were determined by spiral computed tomography scanning. The piriformis and obturator internus muscles can be divided into two and three neuromuscular compartments, respectively. The P_H_ of CINDR of the piriformis muscle was located at 22.61 ± 2.66% of the H line, the P_L_ was at 28.53 ± 6.08% of the L line, and the puncture depth of the piriformis muscle was at 24.64 ± 2.16% of the PP′ line. The P_H_ of CINDR of the obturator internus muscle was at 16.49 ± 1.20% of the H line, the P_L_ was at 10.94 ± 1.09% of its L line, and the puncture depth was 6.26 ± 0.38 cm. These findings may guide the design of the compartmentalized transplantation of the pelvic wall muscles and improve the target localization efficiency and efficacy for injecting botulinum toxin A to treat pelvic wall muscle spasm.

## Introduction

Pelvic wall muscles, including the piriformis and obturator internus muscles, protect the organs within the pelvis and interspersed blood vessels and nerves (Barton 1991; Benzon et al. 2003; Boyajian-O’Neill et al. 2008; Meknas et al. 2003). These muscles permit abduction and external rotation of the hip joint and assist in defecation (Barton 1991; Boyajian-O’Neill et al. 2008). Factors such as stroke, traumatic brain injury, spinal cord injury, and pelvic nerve or pelvic wall muscles subjected to traction may cause pelvic wall muscle spasm (Sammaraiee et al. 2020). Piriformis muscle spasm occurring in addition to restricted hip joint movement can also cause pain in multiple areas, such as the lower back, pelvis, and lower limbs, as well as hinder defecation (Chen and Meng 2020). Long-term spasms of the piriformis muscle can also cause gluteal atrophy (Barton 1991) and leg length discrepancies (Boyajian-O’Neill et al. 2008; Hopayian and Danielyan 2018). Similarly, spasms in the obturator internus muscle can restrict external hip joint rotation, affect the pelvic and perineal areas, cause sciatica, and contribute to difficulties in defecation (Beco and Mouchel 2020; Bhide et al. 2013). Both piriformis muscle and obturator internus muscle spasms can cause painful intercourse (Bhide et al. 2013; Chen and Meng 2020; Schvartzman et al. 2019).

Currently, pelvic wall muscle spasm can be clinically diagnosed via electromyography or vaginal or rectal palpation to identify whether there are nodules in the pelvic wall muscles and relevant painful areas (Aquino-Jose et al. 2020; Papadopoulos and Khan 2004). Treatment options include massage or intramuscular botulinum toxin A (BTX-A) injection to relax the muscles (Fishman et al. 1998; Fowler et al. 2014; Meknas et al. 2003; Santamato et al. 2015). The latter is currently the preferred and effective method (Levesque et al. 2021). When puborectalis syndrome causes pelvic floor outlet obstruction or intractable constipation, obturator internus muscle transplantation has been clinically used for treatment (Farag et al. 1993; Farag 1997). However, there is a risk of loss of function in the donor area after integral muscle transplantation. Dividing the neuromuscular compartments based on the independent primary nerve branches in the muscle and performing compartmentalized muscle transplantation can affect muscle function in both the donor and recipient areas (Letbetter 1974; Lin et al. 2004).

The active site of BTX-A is the motor endplate, and accurately locating its band is key to achieving a curative effect (Amirali et al. 2007). However, collecting fresh specimens and staining the motor endplate band are necessary steps, which make it challenging to determine the exact location of the motor endplate band in the pelvic wall muscles. Nevertheless, studies have confirmed that the position of the motor endplate band is consistent with intramuscular nerve-dense regions (INDR); these can be used as an alternative target for BTX-A (Amirali et al. 2007; Wang et al. 2022). Therefore, this study aimed to reveal the overall intramuscular nerve distribution pattern and neuromuscular compartments of the pelvic wall muscles and localize the exact position and depth (on the body surface) of the center of the intramuscular nerve-dense region (CINDR) to provide anatomical guidance for the treatment of the pelvic wall muscle spasm.

## Materials and methods

### Specimen and ethical approval

Twenty-four adult cadavers (12 males, 12 females) aged 30–75 (66.5 ± 5.3) years without a history of neuromuscular diseases and deformation of the pelvis and hip joints were collected. Among them, 12 (6 males, 6 females) were fixed with formaldehyde for gross anatomy and intramuscular nerve staining; 12 (6 males, 6 females) were cryopreserved and used to localize the CINDR. The specimens were collected and used with consent from the ethics committee of Zunyi Medical University (approval: 2022–1-003). The authors hereby confirm that every effort was made to comply with all local and international ethical guidelines and laws concerning the use of human cadaveric donors in anatomical research. This study was performed in accordance with the ethical standards laid down in the 1964 Declaration of Helsinki and its later amendments.

### Gross anatomy observation, measurement, and reference line design

Twelve formalin-fixed cadavers (6 males, 6 females) were horizontally dissected from the third lumbar vertebra and upper thigh to remove the organs in the pelvic cavity. The specimens were sawed into two halves along the median sagittal plane and carefully dissected. The fascia was removed, allowing for the observation of the origin and insertion of the piriformis and obturator internus muscles, number of nerve branches and their passages, and nerve entry points of the muscles.

To describe the medial and lateral relationship between the CINDR of the pelvic wall muscles and the bony landmarks, as well as the superior and inferior relationship, the following two reference lines were designed: for the piriformis muscle, in the prone position, the curve close to the skin connecting the apex of the coccyx (point A) and the spinous process of the fourth lumbar vertebra (point B) was designated as the longitudinal (L) reference line, and the curve connecting the apex of the coccyx (point A) and the greater trochanter (point C) was designated as the horizontal (H) reference line. For the obturator internus muscle, in the supine position, the curve close to the skin connecting the pubic tubercle (point A) and the medial epicondyle of the femur (point B) was designed as the longitudinal (L) reference line, and the curve between the pubic tubercle (point A) and the greater trochanter was designed as the horizontal (H) reference line.

### Modified Sihler’s intramuscular nerve staining

A previously used method was followed (Wang et al. 2022). The piriformis and obturator internus muscles were immersed in 3% potassium hydroxide and 0.2% hydrogen peroxide solution for 2–3 weeks for depigmentation. This was followed by decalcification in Sihler’s I solution for 4 weeks, staining in Sihler’s II solution for 4 weeks, decolorization in Sihler’s I solution for 3–24 h, and neutralization in 0.05% lithium carbonate solution for 2 h. The muscle was then subjected to gradient glycerol (40, 60, 80, and 100%) for 1 week to attain transparency. Finally, the branches and distribution of the intramuscular nerve were observed under an X-ray film reading lamp, photos were captured, and a pattern map was generated. The photos were opened in Adobe Photoshop CC2019 software (Adobe Company, USA), the INDR were drawn a frame round, and the percentage position of the INDR and CINDR (On the selected INDR, the center point automatically generated by pressing the shortcut key “Ctrl + T” was called CINDR) relative to muscle length and muscle width were measured with CAD software (Autodesk, USA). Subsequently, the nerve distribution pattern map was digitally reconstructed using Adobe Photoshop software, aligning it with the corresponding position on the skeleton, based on proportional calculations.

### Localization of the CINDR in the pelvic wall muscles by spiral computed tomography (CT)

The pelvic wall muscles of 12 frozen cadavers were carefully dissected and exposed, and the muscle belly length and width were measured with a soft ruler. To label the position of the CINDR obtained via Sihler’s staining analysis, 801 Glue (Wenzhou 801 Glue Co., Ltd., China) and medical barium sulfate powder (Sichuan Guangming Medical Equipment Co., Ltd., China) were mixed in the proportion of 4 kg/L and injected into the center of the muscle belly. A silk thread soaked in barium sulfate was sewn on the skin between the body surface landmarks to represent the H and L lines. The specimens were placed in the prone position (for the piriformis muscle) and the supine position (for the obturator internus muscle), respectively, on the 64-row spiral CT (Siemens, Germany), obtained under collimation of 64 × 0.75 mm, slice thickness of 1 mm, pitch of 1:1, automatic tube scanning under milliampere current and voltage of 120 kV, and three-dimensional reconstruction. During the CT scan, the barium sulfate markings helped identify the first white spot corresponding to the CINDR on the cross section of the specimen. With the assistance of the CT scan and perpendicular needle puncture to the skin, the projection points (P) of each CINDR on the body surface (body surface puncture points) were determined under the same bed indicator light. The CT scan process was repeated, and a three-dimensional reconstruction was performed. Under the Syngo system (Siemens, Germany), the total length of H and L lines was measured using a curved measuring tool close to the skin in the cross-section and sagittal plane. The intersections of the vertical line passing through point P with line H and the horizontal line with line L were recorded as points P_H_ and P_L_, respectively, and the lengths between the starting point of the reference line and points P_H_ and P_L_ were labeled H′ and L′, respectively. The values of H′/H × 100% and L′/L × 100% were calculated to determine the percentage position of point P on the skin. On the cross section, point P was projected onto the opposite side of the skin through the CINDR, and this point was marked as P′. Subsequently, the distance between point P and the CINDR (P-CINDR) and the distance between points P and P′ (PP′) were measured. The percentage puncture depth of the CINDR was determined by calculating P-CINDR/PP′ × 100%. For the obturator internus muscle, since a suitable P′ point for performing the clinical measurement could not be found, the absolute depth was measured instead. Based on the fact that the obturator muscle cannot be punctured perpendicular to the skin and requires oblique puncture, the acute angle between the puncture needle and the skin was measured.

### Statistical analysis

All experimental data, expressed as a percentage (mean ± SD)% to eliminate the influence of individual differences in height and weight, were analyzed using the SPPSS 18.0 software (IBM, USA). The measured data were normally distributed. Paired *t*-test was used to compare the data on the left and right sides, and an independent sample *t* test was used to compare the data between men and women, with *P* < 0.05 indicative of statistical difference.

## Results

### Gross anatomical findings

**Fig. 1:**
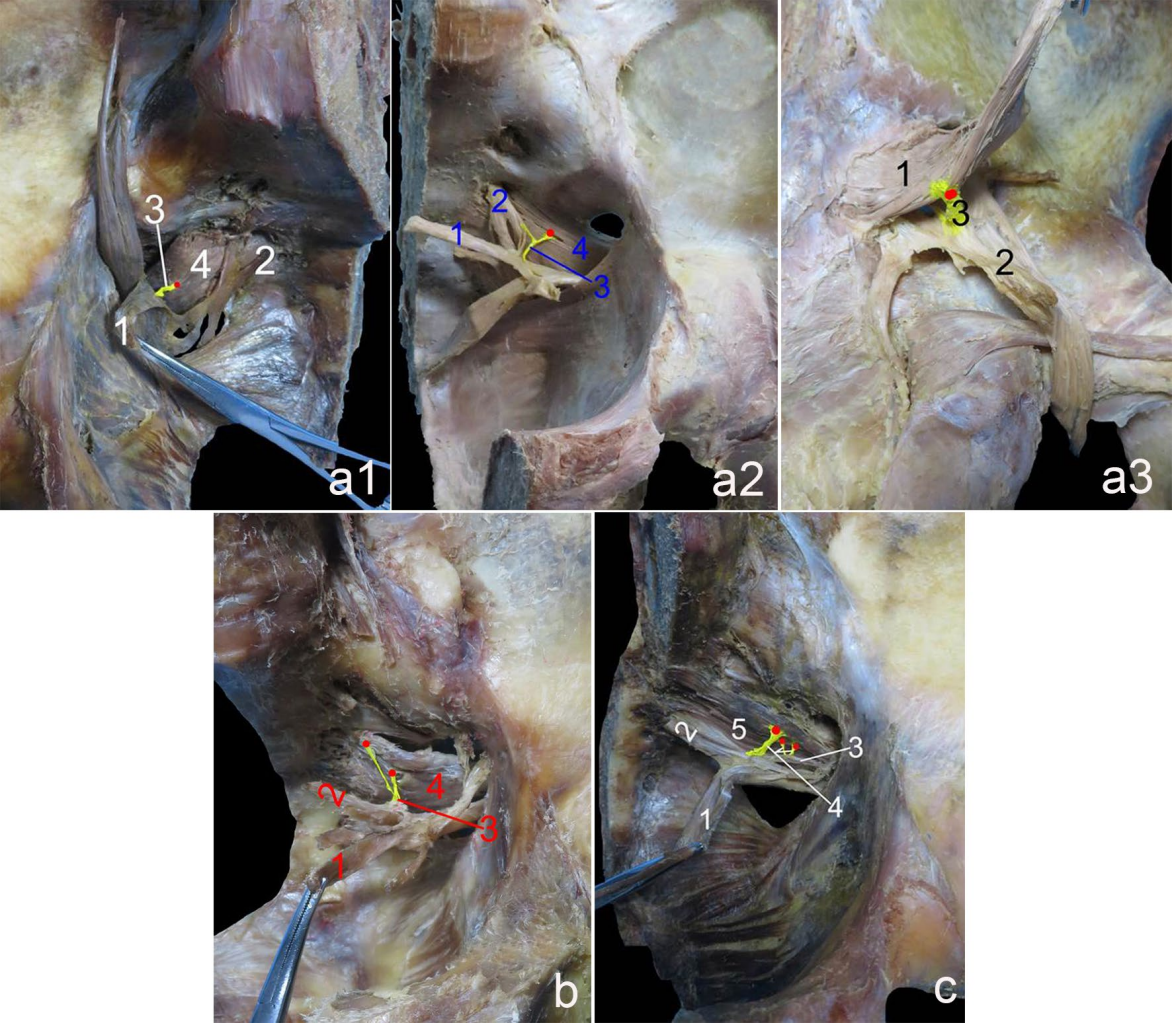
Gross anatomy of piriformis muscle innervation. **a1** A single branch from S1. 1 = S1, 2 = S2, 3 = piriformis nerve muscle branch, 4 = piriformis muscle. The red dot represents the piriformis muscle nerve entry point. **a2** A single branch formed by anastomosis of the S1 and S2 branches. 1 = cut and turned S1, 2 = turned S2, 3 = piriformis nerve muscle branch, 4 = piriformis muscle. The red dot represents the piriformis muscle nerve entry point. **a3** A single branch from the sciatic nerve. 1 = piriformis muscle, 2 = sciatic nerve, 3 = piriformis nerve muscle branch. The red dot represents the piriformis muscle nerve entry point. **b** Two branched types. 1 = S1, 2 = S2, 3 = piriformis nerve muscle branch, 4 = piriformis muscle. The red dots represent the piriformis muscle nerve entry points. **c** Three branched types. 1 = lumbosacral trunk, 2 = S1, 3 = S2, 4 = piriformis nerve muscle branch, 5 = piriformis muscle. The red dots represent the piriformis muscle nerve entry points

**Fig. 2:**
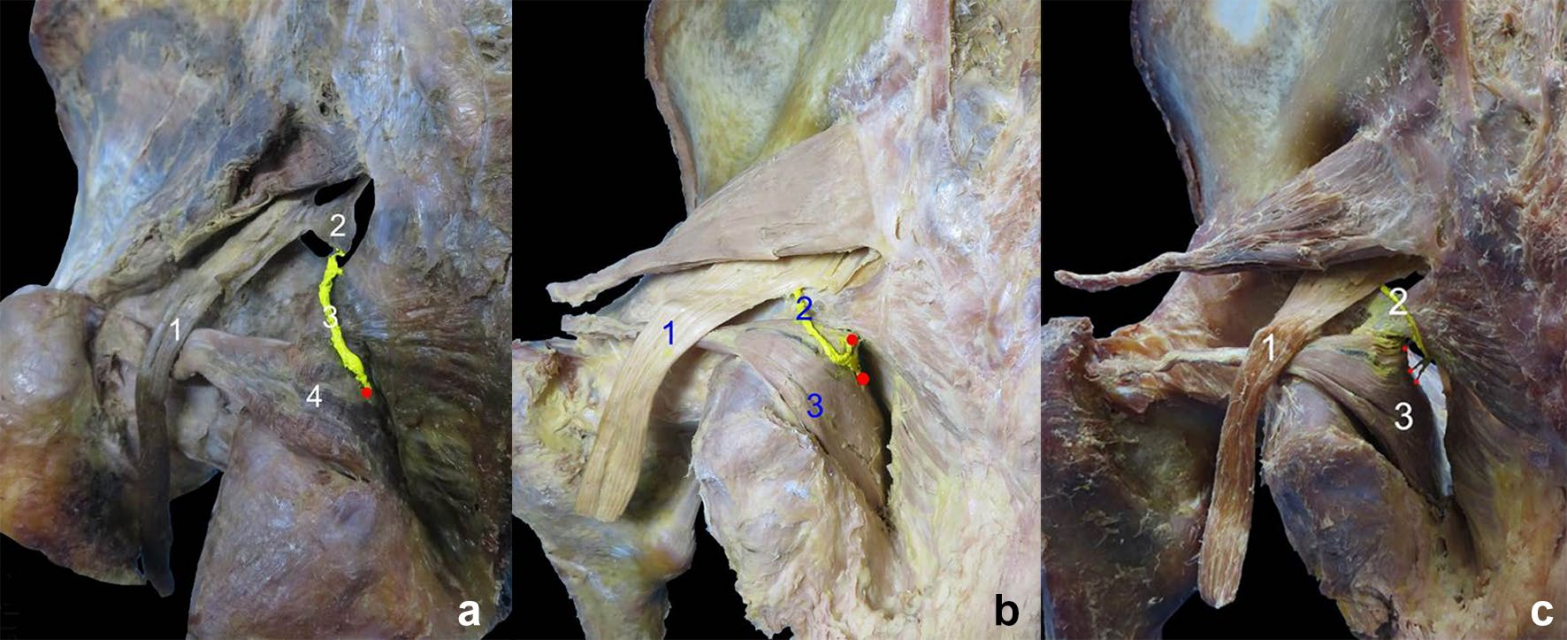
Gross anatomy of obturator internus muscle innervation. **a** A single branch. 1 = sciatic nerve, 2 = sacral plexus branch, 3 = obturator internus muscle, 4 = obturator internus nerve muscle branch. The red dot represents the obturator internus muscle nerve entry point. **b** Two branched types. 1 = sciatic nerve, 2 = obturator internus muscle, 3 = obturator internal nerve muscle branches. The red dots represent the obturator internus muscle nerve entry points. **c** Three branched types. 1 = sciatic nerve, 2 = obturator internus muscle, 3 = obturator internus nerve muscle branches. The red dots represent the obturator internal muscle nerve entry points

Three types of innervation were observed in the piriformis muscles: (1) a single branch emanating either from S1 (37.50%, 9/24) (Fig. 1a1) or from both S1 and S2 before rejoining into a single branch (25.00%, 6/24) (Fig. 1a2) or from the sciatic nerve (4.17%, 1/24) (Fig. 1a3); (2) two branches originating from S2 (29.17%, 7/24) (Fig. 1b); and (3) three branches, one from S1 and two from S2 (4.17%, 1/24) (Fig. 1c). Three types of innervation had also been noted in obturator internus muscles: One branch accounted for 66.67% (16/24) (Fig. 2a); two branches accounted for 20.83% (5/24) (Fig. 2b); and three branches accounted for 12.5% (3/24) (Fig. 2c).

### Sihler’s staining findings

**Fig. 3:**
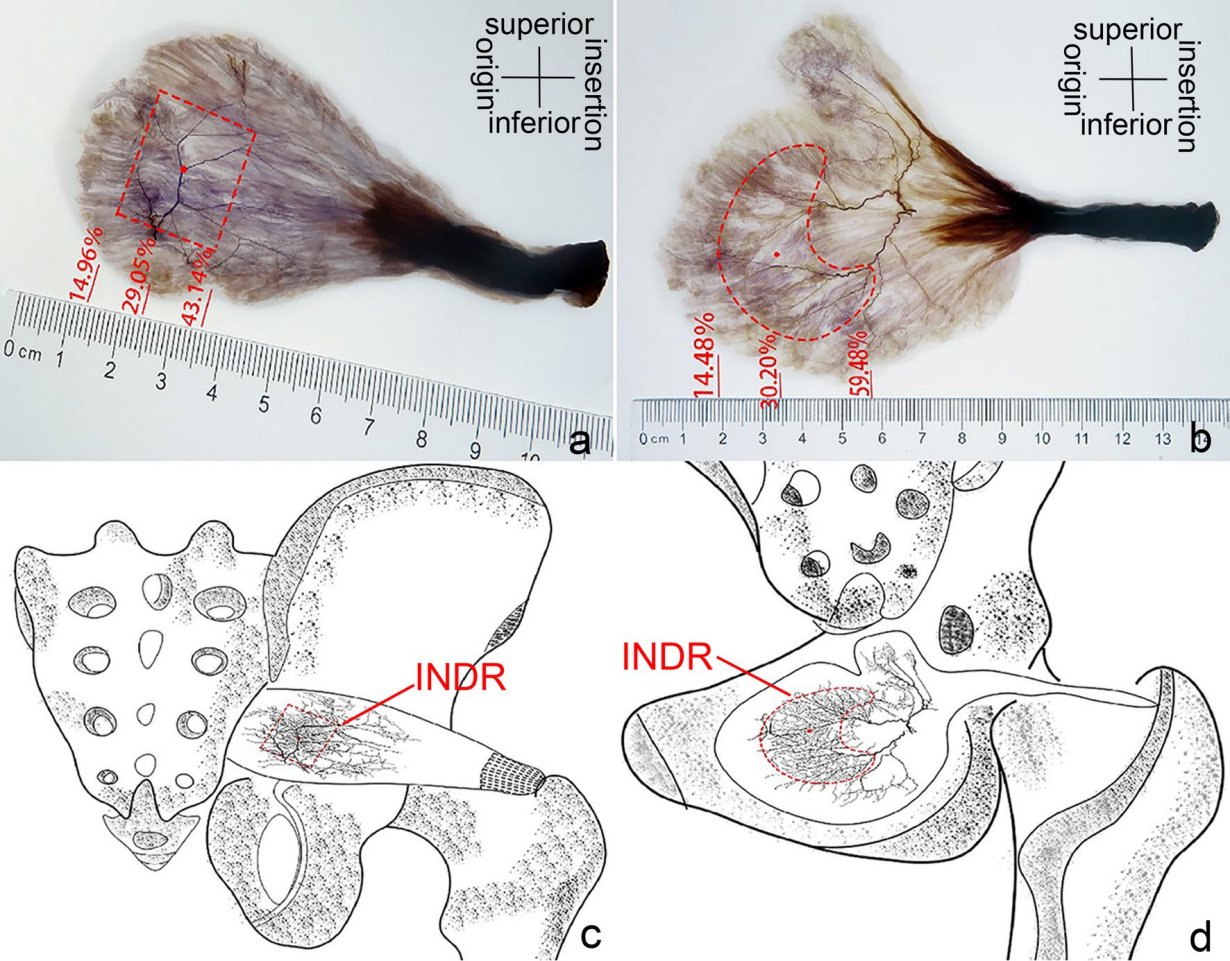
Overall distribution pattern and INDR position of intramuscular nerves in parietal pelvis muscle. **a** Sihler’s staining of the right piriformis muscle. The red box and dot indicate the INDR and CINDR, respectively. **b** Sihler’s staining of the right obturator internus muscle. **c** Intramuscular nerve distribution pattern of the piriformis muscle and intramuscular position of the INDR. **d** Intramuscular nerve distribution pattern of the obturator internus muscle and intramuscular position of the INDR

After entering the muscle, the nerve branches of the piriformis muscle were observed to be divided into two primary branches: the superomedial and superolateral branches. The superomedial branch was relatively thin, with its arborized branches mainly distributed in the muscle’s origin. In contrast, the superolateral branch was thick, and there were more nerve branches at all levels along the way, mainly distributed in the middle and upper parts of the muscle. The nerve branches in the middle and lower parts of the muscle were relatively sparse. Communication existed between the branches of the superomedial and superolateral nerve branches. A rectangular INDR with an area of 5.37 ± 0.32 cm^2^ was formed, located at 14.96–43.14% of the muscle length and 23.93–70.32% of the muscle width (Fig. 3). The CINDR was located at 28.80 ± 0.51% of muscle length and 42.28 ± 0.87% of muscle width. Based on these data, in conjunction with the gross anatomy, the sciatic nerve ran on the deep lateral surface of the INDR. The muscle can be divided into two neuromuscular compartments: the superomedial and superolateral compartments. Upon entering the muscle, the obturator internus muscle nerve branch was observed to divide into three primary nerve branches: superomedial, middle-medial, and inferomedial rami. The branches of the superomedial ramus were relatively sparse and distributed to the accessory head of the obturator internus, which originated from the superior pubic ramus. The inferomedial ramus gave off dense, arborized branches to the inferomedial part of the main body of the muscle belly from the obturator membrane. The dense branches emanating from the middle-medial ramus innervated the upper part of the main body of the muscle belly, while the ending branches were anastomosed with the branches of the inferomedial ramus, forming a lunate INDR (Fig. 3) with an area of 13.33 ± 0.61 cm^2^; its CINDR was located at 30.20 ± 0.25% of muscle length and 36.97 ± 0.82% of muscle width. The obturator internus muscle can be divided into three neuromuscular compartments: superomedial, middle-medial, and inferomedial compartments.

### Spiral CT localization of CINDR

**Fig. 4:**
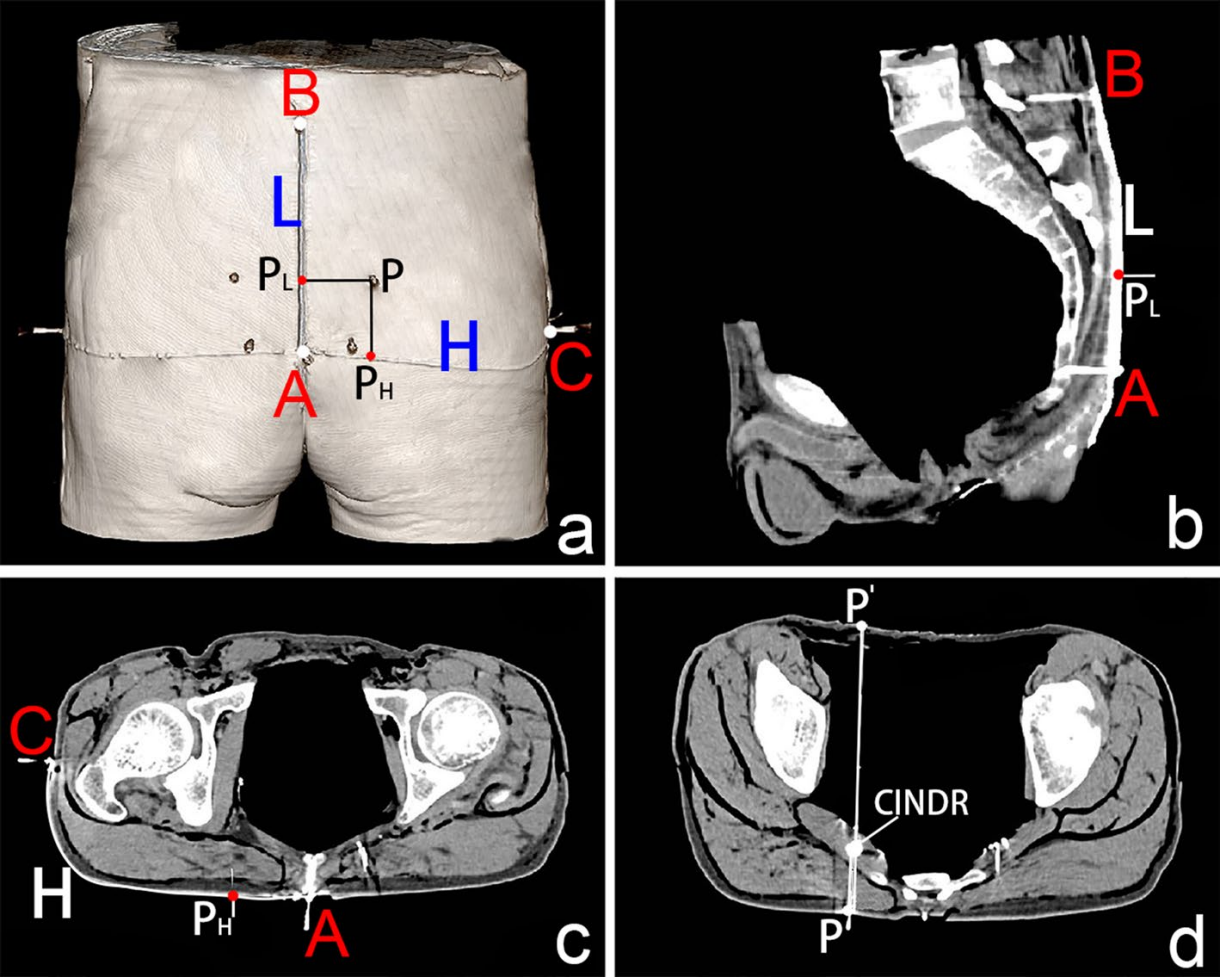
Spiral CT localization image of right piriformis muscle CINDR (male). **a** Body surface projection position of the CINDR and its design reference line. A = apex of the coccyx, B = spinous process of the 4th lumbar vertebra, C = greater trochanter, and P = surface projection point of CINDR on the buttocks. P_H_ point is the intersection point of the vertical line passing through point P and line H, P_L_ point = intersection point of the horizontal line passing through point P and line L, A-P_H_ = H′, A-P_L_ = L′. **b** Measurement of the length of the L and L′ lines on the sagittal section of the fourth lumbar vertebra and coccyx apex. **c** Measurement of the length of the H and H′ lines on the cross-section connecting the tip of the coccyx and the greater trochanter. **d** Measurement of the depth of CINDR on the cross-section through P. The P′ point is the point where the P point passes through the CINDR point and projects onto the opposite side surface

**Fig. 5:**
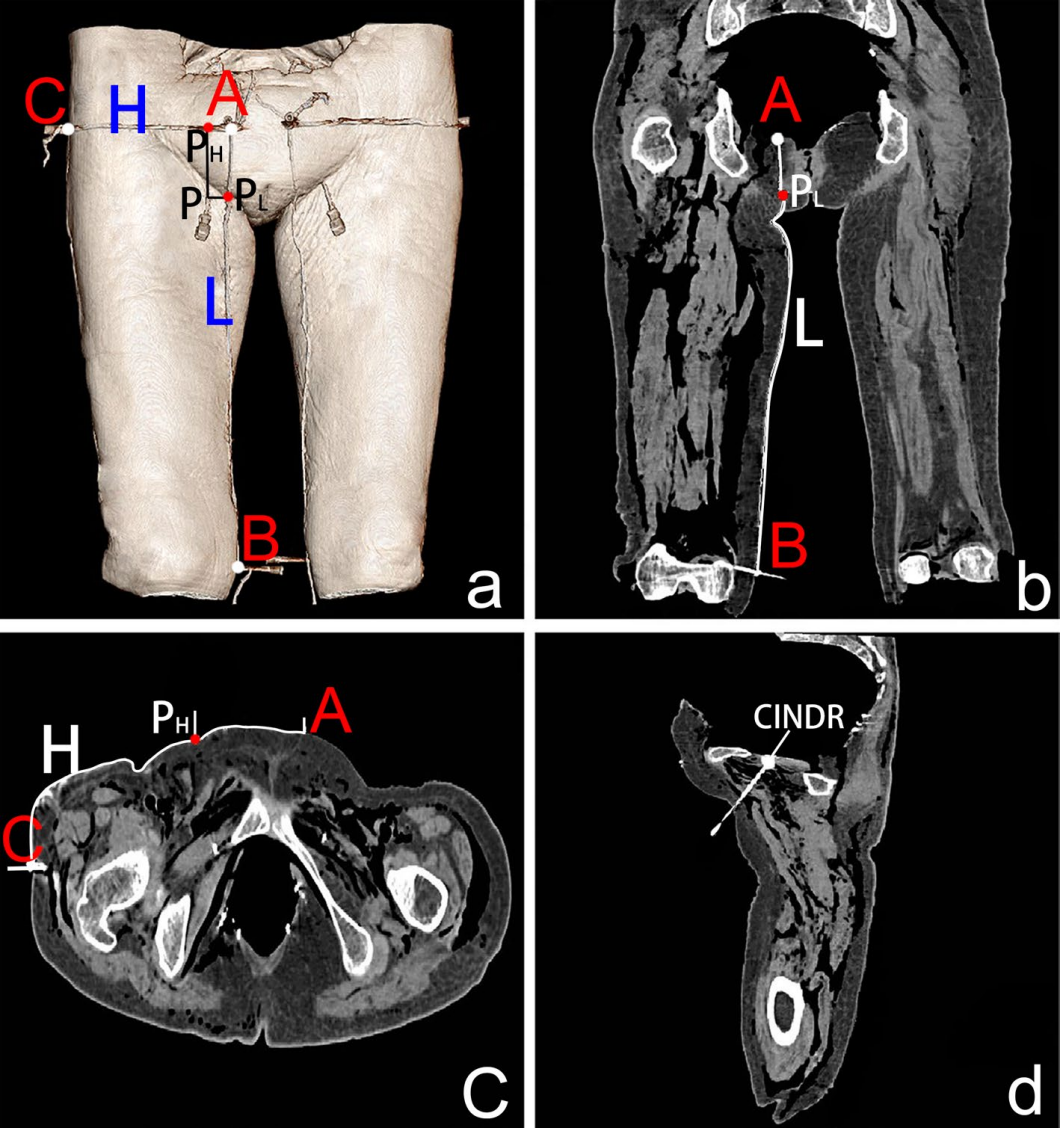
Spiral CT localization image of right obturator internus muscle CINDR (female). **a** CINDR body surface projection position and the designated reference line. A = pubic tubercle, B = medial epicondyle of femur, C = greater trochanter, P = projection point of CINDR in front of the thigh. P_H_ is the intersection of a vertical line through P with line H, P_L_ = intersection of a horizontal line through P with line L, A-P_H_ = H′, A-P_L_ = L′. **b** The length of the L and L′ lines was measured through the pubic tubercle and coronal section of the medial epicondyle of the femur. **c** The length of the H and H′ lines was measured on the cross-section of the pubic tubercle and the greater trochanter connection. **d** The depth of the CINDR was measured through the sagittal plane of the P point

The spiral CT localization images of the CINDR of the muscles of the pelvis wall are shown in Figs. 4 and 5. The P_H_ of the CINDR of the piriformis muscle was at 22.61 ± 2.66% of line H, P_L_ was at 28.53 ± 6.08% of line L, and the puncture depth was at 24.64 ± 2.16% of the PP′ line. In front of the thigh base, when the puncture needle was 45°–55° from the body surface, it was easy to penetrate the CINDR of the obturator internus muscle successfully, and the puncture point was far from the femoral and obturator vessels and related nerves. In this way, the P_H_ of the CINDR of the obturator internus muscle was at 16.49 ± 1.20% of its H line, and P_L_ was at 10.94 ± 1.09% of its L line. The puncture depth was 6.26 ± 0.38 cm. There was no significant difference between males and females or between the left and right sides (Tables 1 and 2).

**Table 1:** P_H_ and P_L_ positions and CINDR of left and right pelvic wall muscles on the H and L lines, respectively (mean ± SD)

CINDR	P_H_ on line H (H′/H%)				P_L_ on line L (L′/L%)				Depth of CINDR (piriformis, %; obturator internus, cm)			
	Left (*n* = 12)	Right (*n* = 12)	*t*	*P*	Left (*n* = 12)	Right (*n* = 12)	*t*	*P*	Left (*n* = 12)	Right (*n* = 12)	*t*	*P*
CINDR of the piriformis	22.68 ± 2.76	22.53 ± 2.67	–1.09	0.34	28.81 ± 1.86	28.25 ± 1.73	2.15	0.15	24.75 ± 2.56	24.53 ± 1.79	0.72	0.48
CINDR of the obturator internus	16.50 ± 1.21	16.48 ± 1.13	0.04	0.93	10.93 ± 1.14	10.95 ± 1.08	–0.23	0.82	6.28 ± 0.30	6.25 ± 0.46	0.35	0.73

*CINDR *center of the intramuscular nerve dense region

**Table 2:** Comparison of the P_H_ and P_L_ positions on the H and L lines and the depth of CINDRs in pelvic wall muscles between males and females (mean ± SD)

CINDR	P_H_ on line H (H′/H%)				P_L_ on line L (L′/L%)				Depth of CINDR (Depth of CINDR (piriformis, %; obturator internus, cm)			
	Males (*n* = 12)	Females (*n* = 12)	*t*	*P*	Males (*n* = 12)	Females (*n* = 12)	*t*	*P*	Males (*n* = 12)	Females (*n* = 12)	*t*	*P*
CINDR of piriformis	23.59 ± 1.76	21.63 ± 2.01	1.21	0.34	29.36 ± 2.56	27.70 ± 1.89	0.85	0.39	25.67 ± 1.78	23.61 ± 2.06	2.64	0.53
CINDR of obturator internus	17.21 ± 1.92	15.77 ± 3.61	1.38	0.25	11.01 ± 1.26	10.87 ± 0.95	0.31	0.17	6.47 ± 0.31	6.05 ± 0.33	3.20	0.53

*CINDR* center of the intramuscular nerve dense region

## Discussion

Pelvic wall muscle spasm can lead to limited hip mobility, urinary and reproductive dysfunction, pain (Barton 1991; Bhide et al. 2013; Chen and Meng 2020; Karp et al. 2019; Schvartzman et al. 2019), and sciatica (Balius et al. 2018; Park et al. 2020). Clinically, it can be treated by blocking the release of acetylcholine from the presynaptic membrane to the motor endplate, resulting in muscle relaxation (Bhide et al. 2013; Chen and Meng 2020). Considering the lack of information regarding the motor endplate band of the pelvic wall muscles, the position of the INDR is consistent with that of the motor endplate band, and the therapeutic potential of obturator internus muscle transplantation in addressing conditions like pelvic floor outlet obstruction or intractable constipation resulting from puborectalis syndrome, it becomes crucial to gain a comprehensive understanding of the innervation of the pelvic wall muscles, accurately localize the position of the INDR of the pelvic wall muscles, and explore the related neuromuscular compartments. This will be highly significant in providing direction for treating the pelvic wall muscle spasm.

Numerous studies have investigated the origin of extramuscular innervation in the muscles of the pelvic wall, and most of the studies indicated that the piriformis muscle is innervated by branches of S1 and S2 nerves (Boyajian-O’Neill et al. 2008; Fishman et al. 1998). However, another study suggests that the piriformis muscle is innervated by L5, S1, and S2 nerves (Iwanaga et al. 2019). In this study, the piriformis muscle in one individual was found to be innervated by a branch of the L5 nerve (i.e., a branch of the sciatic nerve). Additionally, Balius et al. (2018) described that the obturator internus is innervated by a branch of the sacral plexus, which runs on the deep surface of the sciatic nerve. The results of the present study are consistent with these previous study findings.

Regarding research on the intramuscular innervation of the pelvic wall muscles, Yi et al. (2021) showed the intramuscular nerve distribution pattern of the piriformis muscle via the modified Sihler’s staining technique to determine the ideal injection area for BTX-A treatment of the piriformis syndrome. The results showed that the nerve entry point of the piriformis muscle was located in one-fifth of the area connecting the lateral edge of the sacrum to the greater trochanter, and the intramuscular nerves were densely distributed in one-fifth to two-fifths of the area between the lateral edge of the sacrum and the greater trochanter. In that study, the lateral edge of the sacrum was used as a reference landmark, which had a large scope and was not a specific bony landmark. By comparison, the present study showed the arborized intramuscular nerve distribution region more clearly. With the help of bony landmarks and spiral CT scanning, the body surface puncture position and CINDR depth were accurately localized, and the division of neuromuscular compartments was simultaneously explored. However, for the obturator internus muscle, the intramuscular nerve distribution could not be reported.

There have been some research reports on the localization of nerve block targets in the pelvic wall muscle. For example, Wan-Ae-Loh et al. (2020) measured the distance between the posterior superior iliac spine and the ischial tubercle, the posterior superior iliac spine and the greater trochanter, and the ischial tubercle and the greater trochanter to determine the boundary of the piriformis muscle and clarify the anatomical relationship between the piriformis muscle, the ischial nerve, and the gluteal fold. Through rectal palpation, Abbott (2009) determined painful nodules on the piriformis muscle at 4–5 and 7–8 oclock positions. Bevilacqua Alén et al. (2016) and Aquino-Jose et al. (2020) performed an ultrasound-guided intramuscular drug injection into the piriformis. Gupta et al. (2017) injected dye through the vagina in fresh cadaver specimens and found that the obturator internus muscle was located at the 1 and 11 o’clock positions. There is also a report on obtaining the blocking site of the obturator internus muscle by everting the buttock, finding the ischial tuberosity, and touching the contraction of the obturator internus muscle in the direction of the greater trochanter (Karp et al. 2019). However, these methods are unsuitable to locate INDRs (i.e., the true location of effective targets). Transvaginal injection into the obturator internus and piriformis muscles carries risks of vaginal infection and ischiorectal abscess (Brueseke and Lane 2012).

Nerve endings often form motor endplates in the middle of muscle fibers, and the motor endplate band is formed in the middle part of the muscle belly of the spindle muscle; therefore, BTX-A is often injected into the middle part of the muscle belly (Amirali et al. 2007). However, owing to their different architectures, differently shaped muscles have motor endplate bands in different positions, which may not be located in the middle of the muscle belly (Wang et al. 2022). The results of this experiment suggest that the INDR and CINDR of the obturator internus and piriformis are not located in the middle part of the muscle belly; if BTX-A is injected into the middle part, it may not produce the desired results. Although ultrasound guidance can identify muscle contour and depth, it does not identify tiny INDR. In this study, the corresponding CINDR locations were labeled with barium sulfate, and then the puncture location and depth of the body surface were determined by spiral CT scanning with the help of bony landmarks, which was conducive to improving target location identification and, consequently, BTX-A injection efficiency and efficacy.

The efficacy of a BTX-A injection is closely related to finding the exact target location. If the injection site is 5 mm from the motor endplate, the antispasmodic effect will be reduced by 50% (Tang et al. 2018). In some studies, up to 60 units of BTX-A was injected into the obturator internus muscle under electromyography guidance to treat spasms (Chen and Meng 2020). Such large-dose injections may cause side effects such as excessive muscle relaxation, fibrosis, and antibody generation. Therefore, accurate and appropriate BTX-A injection to the target site is essential. Studies have shown that 1 unit of BTX-A can infiltrate muscle tissue 1.5–3 cm^2^, and 2.5–5 units can diffuse into 4.5 cm^2^ (Borodic et al. 1994; Yu et al. 2023). According to these theories and the results of this experiment, the INDR areas of the piriformis and obturator internus muscles are 5.37 ± 0.32 cm^2^ and 13.33 ± 0.61 cm^2^, respectively. If the injection area is accurate, only 3.4–3.8 and 8.5–9.3 units of BTX-A need to be injected into each side.

Letbetter (1974) found that the muscle fibers innervated by the first branch of the intramuscular nerve are tightly integrated, and this part of the muscle can be used as a sensorimotor compartment. Based on the modified Sihler’s staining method, which can be used to determine the overall distribution pattern of intramuscular nerves visible to the naked eye (Wang et al. 2020), the present study demonstrated the distribution pattern of the intramuscular nerves of the pelvic wall muscles to reveal whether these muscles could be compartmentalized. Our findings suggest that these two pelvic wall muscles can be divided into two neuromuscular compartments. Therefore, in clinical cases where obturator internus muscle transplantation is performed to treat anal outlet syndrome, it is advisable to consider performing neuromuscular compartment transplantation based on the required muscle force and dynamic range of the recipient area. This approach facilitates normal innervation and optimal functional recovery of the recipient area while minimizing complete damage to the donor area. In cases of piriformis syndrome caused by the compression of the sciatic nerve by the piriformis muscle, which can lead to walking abnormalities, if it becomes necessary to release the sciatic nerve by partially cutting the piriformis muscle fibers, it is recommended to prioritize cutting the relatively smaller superomedial compartment of the muscle. This approach helps reduce damage to the overall integrity of the muscle. Furthermore, it is advised to separate and excise the sparsely innervated regions located near the middle and lateral parts of the muscle. This step helps avoid unnecessary damage to the intramuscular nerve branches, thus preserving their functional integrity.

Overall, this study revealed the neuromuscular compartment of the pelvic wall muscles through Sihler’s staining, providing a morphological basis for the compartmentalized material design in clinical surgical muscle transplantation. Identification of INDR and labeling of CINDR with barium sulfate, spiral CT scanning, and three-dimensional reconstruction helped establish the geometric relationship between the CINDR and body surface landmarks. This enabled the determination of the body surface projection location and depth of CINDR, which can provide a morphological guidance for precisely localizing BTX-A injection target points in treating pelvic wall muscle spasm. However, it is important to acknowledge the limitations of this study. First, this study did not explore potential racial differences, and the findings may not be generalizable to diverse populations. Second, this study solely focused on anatomical investigations and did not directly assess the therapeutic effects of target blockade and neuromuscular compartments. Further clinical research is necessary to validate the clinical benefits and outcomes associated with these findings.

## Data Availability

The data from this study will be made available upon reasonable request to the corresponding author.
